# Experiences of person-centered care for sundown syndrome among nurses and nurse aides in dementia special care units: a qualitative study

**DOI:** 10.1186/s12912-023-01598-x

**Published:** 2023-11-17

**Authors:** Su-Fei Huang, Bow-Yin Wang, Jung-Yu Liao

**Affiliations:** 1 Department of Intelligent Technology and Long-Term Care, MacKay Junior College of Medicine, Nursing, and Management, No.92, Shengjing Rd, Taipei, 11260 Taiwan; 2 St. Joseph Home for Alzheimer’s Disease and Related Dementia, No.11, Lane 125, De Chang St, Taipei, 10867 Taiwan; 3 Department of Health Promotion and Health Education, National Taiwan Normal University, No.162, Sec. 1, He-ping East Road, Taipei, 10610 Taiwan

**Keywords:** Person-centered care, Dementia, Nurse, Qualitative research, Focus Groups, Dementia special care units

## Abstract

**Background:**

To explore the response and management experiences of nurses and nurse aides in dementia special care units when caring for residents with sundown syndrome based on the person-centered care model.

**Methods:**

Focus group interviews were conducted among nurses and nurse aides from four dementia special care units that have been accredited by the Ministry of Health and Welfare in Taiwan. Content analysis was used for data analysis.

**Results:**

The 29 nurses and nurse aides were recruited to participate in the study. Analysis of interview content revealed six themes, identifying the intra-individual, inter-individual, and organizational dimensions. The central topic was commitment. Under the umbrella of commitment, six themes including self-preparation, non-suppression, diversion, pacification, continuity of meeting, and collaboration, which had 18 subthemes, emerged as responsive care practices for person-centered care when supporting residents with sundown syndrome.

**Conclusions:**

The findings provide responsive care practices based on person-centered care for people living with dementia who develop sundown syndrome. The study can inform practices for quality of care for dementia in long-term care institutions and contribute to the development of materials for nursing training and education.

**Supplementary Information:**

The online version contains supplementary material available at 10.1186/s12912-023-01598-x.

## Introduction

It is estimated that at least 57 million people living with dementia (PLWD) in 2019, and this number is expected to triple by 2050 [1]. Sundown syndrome (SS) is a common phenotype in PLWD, which is associated with a considerable magnitude of care distress. A scoping review study based on older adults aged ≥ 60 years found that the frequency of SS varied from 2−82% [2]. It can be considered a periodic delirium-like syndrome, including confusion, anxiety, wandering, climbing, agitation, and aggressiveness [3]. Generally, SS is observed in PLWD after late afternoon and may persist for several hours, with a high frequency of neuropsychiatric symptoms [4]. Despite a lack of formal recognition of and clinical definitions for SS, it has been associated with mental and behavioral symptoms and cognitive impairment [2], possibly due to light’s critical role in complex mechanisms [5]. Other influencing factors may include impaired circadian rhythms, environmental and social factors, and cognitive impairment [3]. Therefore, PLWD with SS indicates an emergence of or increase in certain behavioral and psychological symptoms, increasing the challenges of caring for affected persons.

The person-centered care (PCC) model for PLWD has been advocated for several years; it is widely attributed to Kitwood’s framework grounded in personhood and involves aspects of basic psychological needs (e.g., comfort and attachment), addressing malignant social psychology, and positive person work (e.g., negotiation and collaboration) [6–8]. PCC emphasizes tailored and individualized care, taking into account the perspectives of PLWD, and prioritizes a strong foundation of active psychosocial support [9]. Some commonalities among models and practices of PCC for PLWD have been found, and one example among them is a framework encompassing the elements of value, individualization, perspective, and social psychology [10]. Numerous studies have explored experiences of institutional care providers adopting a PCC approach for PLWD in Europe and North America, regarding PCC as a crucial element in the care and interventions [11]. However, they have focused more on specific programs at the individual level (e.g., life stories) [12] or at organizational-level changes (including therapeutic environments) [13, 14], and less on real scenarios of PCC in daily care at the intra-individual, inter-individual, and organizational levels. Despite recommendations to implement PCC for PLWD across all care settings [15], its practical application in daily care is still limited. To the best of our knowledge, the application of PCC within an Asian cultural context remains to be explored and a subject requiring immediate attention is care for PLWD with SS.

In Taiwan, various services including home care, community care, and institutional care are part of efforts to address dementia care needs as stipulated by the long-term care policy (2.0). Dementia special care units (DSCUs) have gradually expanded in special accommodation institutions that primarily care for ambulatory residents with moderate-to-severe dementia. Nurses and nurse aides collaborate closely in providing care in DSCUs. PLWD receive their care from nurse aides who are responsible for caregiving tasks and directly addressing the care needs of PLWD. Nurses not only assist with nursing care but also play a supervisory role. However, nurses or nurse aides may be prone to conflict and could experience burnout, as they relentlessly provide care from morning to evening and must deal with the chaos resulting from SS. For example, PLWD who experience SS face particular challenges with regard to their daily routines (e.g., dressing) [16] and medication adherence [17]. Even professionally trained care providers find it difficult to cope with SS presentations [18].

There is an urgent need to support cooperation between nurses and nurse aides when implementing the PCC model for PLWD to address conflicts, particularly when dealing with the challenging behaviors exhibited by PLWD during handover, when they are fatigued and under high stress after a day’s work. Therefore, this study aimed to increase the understanding of the response and management experiences of DSCU frontline nurses and nurse aides when their residents develop SS based on the PCC model.

## Methods

### Participants

Nurses and nurse aides were recruited from the participating DSCUs in the Greater Taipei Area that have been accredited by the Ministry of Health and Welfare. The inclusion criteria were as follows: (1) having experience caring for SS, (2) aged 20 years and above, (3) no sex restriction, and (4) willingness to participate in the study.

The research team explained the study purpose and processes to the DSCU chairs. After receiving their approval for the study, the recruitment advertisement describing the study and the main topic (Table 1) discussed in the focus group interview was posted on the bulletin board of each participating unit. Finally, four focus group interviews were conducted with 29 nurses and nurse aides; data saturation was reached following the fourth focus group interview as no new perspectives were generated.

### Focus group interview process

Four focus group interviews were conducted in the participating DSCUs (anonymized: Units A, B, C, and D), with 5−8 participants per group [19]. For the interviews, we recruited the eligible nurses and nurse aides between October and November 2019. A large volume of interview data with breadth and depth was collected under the guidance of the interviewer [20]. The main topics were covered in the open-ended questions containing interview prompts (Table 1), which were part of the interview guide used by the moderator. The interview prompts were developed based on a literature review and revised by three experts in geriatric psychology.

**Table 1:** Interview guide used in the focus group, with main topics, questions, and prompts

Main topic	Questions and prompts
• SS symptoms	1. Based on your care experience, what SS symptoms do the residents have? 2. What are the common symptoms of SS?
• Person-centered dementia care: Individual dimension	1. How do you care for a resident when he or she shows problematic behavior due to SS? 2. What symptom of SS causes the most problem for you during the care process? Why?
• Person-centered dementia care: Multiple dimensions	1. How do you care for two or more residents when they show SS symptoms at the same time? 2. What kind of assistance do you expect when caring for older adults with SS? 3. How does the unit manage residents when they show problematic behavior due to SS?

SS = Sundown syndrome

Each focus group interview (lasting approximately 1–1.5 h) was arranged by a contact person in each participating unit; each interview started with an opening introduction and the completion of a general personal information form. The moderator of the focus group interviews was the first author, who has extensive knowledge and expertise on matters related to PLWD and their caregivers and a decade of teaching experience in dementia care. To ensure the moderator’s neutral and catalytic role in the interviews, an immediate review and transcription of the interviews were conducted after each focus group.

During the interviews, the moderator asked open-ended questions from the interview guide and ensured that the participants had equal opportunities to share their perspectives. First, the participants spoke about the SS symptoms they have witnessed in their care experience and then shared their perspectives of and strategies for person-centered dementia care in different scenarios. Moreover, the moderator encouraged all contributions to the discussion, and the similarities and differences between various perspectives were compiled. Collaboration among participants with varied perspectives facilitated greater interaction within the focus group. Furthermore, a discussion was held based on the participants’ statements, and “restatement” was employed to ensure accurate understanding of participants’ responses.

### Data processing and analysis

Each focus group interview was recorded and transcribed to obtain a description of the care experience. Content analysis was used. The first step was to review the interview content repeatedly and develop a coding framework. The research members independently reviewed the same set of interview transcripts and highlighted any key points of interest as initial ideas, which were then shared and discussed in a team meeting to reach a consensus. The second and third authors continuously performed data comparisons and compared the data using the same codes. The first author made a decision in case of any inconsistencies. Thereafter, they reconfirmed whether data with the same code contain the same concepts and whether the data were appropriate [21]. Subsequently, the four interview transcripts were coded and classified into frameworks to obtain the main themes. The data reduction process was performed for all analysis units, including the themes and subthemes as well as the central topic. An example of the data analysis process is presented in Additional Table 1 [additional file]. Quotes were selected to present participants’ responses regarding SS care. All authors have extensive experience in dementia care and research, and all participated in discussions and continuous analysis, confirmed the themes, and ensured that the data were saturated [22].

Next, the dependability, credibility, and transferability of qualitative studies were conducted. We periodically reviewed the data analysis context, process, and results to reconfirm that the data were reliable and avoided excessive personal bias (Dependability). Subsequently, some interviewees were randomly selected to rectify facts or transcription errors (Credibility). This study had a specific context and we placed restrictions on the inference of study results to ensure the rigor of the study (Transferability). Finally, we summarized the interview data analysis results to compile the study results and interpret the content.

### Ethical consideration

This study was approved by the Research Ethics Committee of National Taiwan University (201907ES030). The researcher explicitly explained the purpose and procedures of the study to all the participants, and their signed informed consent was obtained to confirm that they fully understood and consented to participate in the study.

## Results

### Interviewees’ background characteristics

Of the 29 participants, eight (27.6%) were nurses and 21 were nurse aides (72.4%). There were 27 women (93.1%) who participated in this study. The mean age was 51.6 years (SD = 9.29), and the mean level of work experience was 9.07 years (SD = 5.39). Among the participants, the four focus groups included two nurses and three nurse aides in Unit A (age range: 53–61 years), two nurses and six nurse aides in Unit B (age range: 42–60 years), two nurses and six nurse aides in Unit C (age range: 24–64 years), and two nurses and six nurse aides in Unit D (age range: 26–63 years).

### SS population care problems and causes

Analysis of interview content revealed that common challenges and scenarios in the residents with SS include: (1) Wandering: wandering or continuous walking; (2) Seeking escape: walking toward elevators and wanting to go out; (3) Continuous clamoring to return home/look for family members; (4) Repetitive speech or behavior: asking the same question repeatedly or rummaging; (5) Hallucinations: Visual and auditory hallucinations; (6) Delusion: delusion of theft, persecutory delusion; (7) Sleep problems: circadian rhythm disruption; (8) Inappropriate sexual needs; (9) Emotional problems: anxiety, shouting; (10) Aggressive behavior: cursing, physical violence (toward objects or people); (11) Eating disorders: binge eating, refusal to eat, eating wrong foods; (12) Bathing: refusing guidance to bathe; and (13) Toileting problems: frequent toileting, urination outside of the toilet.

### Practice of PCC for PLWD with SS

**Fig. 1:**
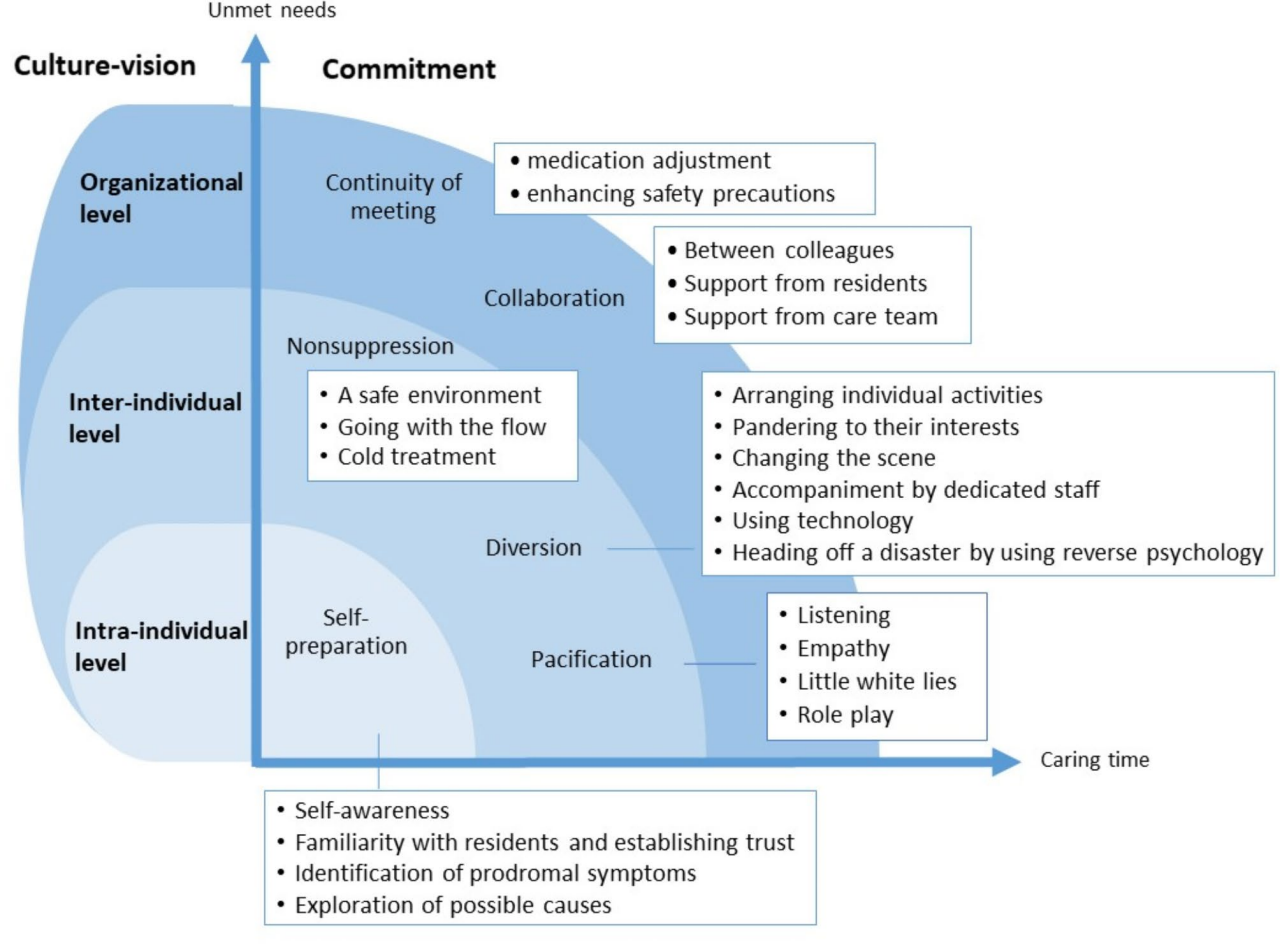
Themes and sub-themes illustrating the practice of person-centered care for older residents with sundown syndrome

Through qualitative content analysis, the central topic was commitment while six themes and 18 subthemes emerged as responsive care practices, identifying the intra-individual, inter-individual, and organizational dimensions (Fig. 1). As dementia progresses, clinical care practices for PLWD with SS grow increasingly complex, leading to greater unmet needs and extended caregiving time for nurses and nurse aides. The practical strategies related to PCC must be shifted from the initiation of self-preparation at the intra-individual level to strategies at the inter-individual level, involving pacification, non-suppression, and diversion techniques with residents with SS. The team needs to be mobilized for additional support at the organizational level if the caring scenarios the nurse and nurse aides face become increasingly complex. Table 2 shows the participant quotes with the themes and subthemes.

**Table 2:** Quotes for themes and subthemes

Theme 1 Self-preparation: subthemes and quotes	
Self-awareness	The resident has the SS, which may also be induced in me! (B339–340). Often, residents may become confused. When I hear that an older woman is searching a scarf, I think: “Oh no, here she comes again, and know that today is going to be a bad day.” (B342–343). Control my emotions and do not get angry! Do not be affected by older residents. I cannot let myself be disturbed by their emotions, even when they create disturbance. (B273–274).
Familiarity with residents and establishing trust	When an older resident shows emotions or the SS occurs, only staff with whom he/she is familiar can make him/her feel safe and at ease. (A196–197).
Identification of prodromal symptoms	If an older resident acts strange, we should not ignore or pretend to not him/her, as it is difficult to pacify him/her when his/her emotions appear. This will cause a chain reaction and result in difficulty in subsequent management. (B289–292). When you can predict the appearance of the SS in a resident, arrange for him/her to rest or partake in an activity beforehand. (A173–175).
Exploration of possible causes	(Restless behavior) I think of what he/she wants to express. Is it due to a physical condition, like wanting to go to the toilet, hunger, dry mouth, or other problems that cause restless behavior? I will first satisfy his/her physiological needs. (C017–020).
Theme 2 Pacification: subthemes and quotes	
Listening	When encountering an anxious or restless resident, I first hold his/her hand, ask him/her to sit down together, and ask what happened. How was he or she? (C022–031). When an older resident shows emotions, or he/she is anxious or crying, I act with consideration and affection to pacify him/her! I listen to ascertain what he/she wants. (B156–159).
Empathy	(Resident who asks for her son every afternoon) Every time, she talks to me as I pacify her. I say, “Your son has found someone to take care of him and you need not worry. Please sit here and I will take you to find him later.” She says, “Later you must take me to him else I will cry.” I say, “You cannot cry. You have to sit here and wait.” She then sits down and forgets about it. Others say, “Your son is not a child but adult so do not worry about him.” However, in her memory, the son is at a stage where he requires her care… (A140–146).
Little white lies	When an older resident requests to go home, I say, “Please wait for a while because the unit is on a mountain. We do not have a vehicle to transport you home. Later I will call your son and ask him to come fetch you.” The resident says, “Really? Are you going to call him?” I reply, “You have to give me some time. I must go to the office to make a call. I will call him and ask him to come fetch you.” This pacifies the resident who forget the conversation after a while. (A017–025). (Looking for family members) I say: “Oh no! The vehicle just left. Why are you looking for your family members? Why didn’t you say so earlier? You would have to sleep here tonight. There will be free transport tomorrow morning and the first bus can send you home.” This was used to pacify residents. (B078–081).
Role play	(Looking for exit, requesting to go home) We pretended to be her family members and called her to tell him that we were on the way to the institution. We used this method to make her think that her family members had called her, and she would accept any reason given by her fake family members, such as a car breakdown. (B093–100). An older resident used to rush to the elevator saying that his daughter had called. So, we asked a female staff from another department to call him. After talking to the impersonator, he forgot about rushing to the elevator. (D045–046). An older resident with persecutory delusions kept thinking that we were stealing his money and chased us. We would first hide in a room, with masks on, and then remove the masks and come out. He would think we were other people, and this pacified him. (D059–062).
Theme 3 Diversion: subthemes and quotes	
Arranging individual activities	When the resident becomes restless, he/she will find things to do. These activities include asking older people to sort beans. A few older residents may need people to accompany them, sing, watch television, or want to perform certain activities quickly (B440–443).
Pandering to their interests	One method of diverting attention from older residents is to identify things that they like (B060–061). Ascertain their preferences and give it to them. Assuming that they enjoy music, we first allow them to listen to music. In case they like to watch television or eat, we pander to their likes (D042–044). In case they keep searching for an exit, wanting to go home, and wandering around anxiously, we first identify things they like, such as food or chatting, to distract their attention (C010–011). There are certain older residents who like cash; we ask their family members to bring stacks of fake currency notes for them to count (E313).
Changing the scene	I take the resident away from the scene (public space) and to his/her room. There, I spend 5–10 min to distract him/her by folding clothes or writing. After his actions become stable, I leave the room, periodically returning to see what he/she is doing (C100–104). (Management of visual and auditory hallucinations) First, we guide him to another place, away from that environment, and then help him/her manage the emotion. This helps him/her forget about the hallucinations (D052–055).
Accompaniment by dedicated staff	At night, we have time to accompany the resident. For example, in case he/she wants to pack clothes, I help him/her pack. The accompaniment process can divert his/her attention; I ask him/her when did he/she buy these clothes? Which clothes does he/she like? This can divert his/her thoughts of returning home (B183–191).
Using technology	(When a resident is restless and wants to find his/her son) I tell him/her that the son is currently busy. I also show him/her a video of the son, which may not be effective, but it diverts his/her attention, resulting in slight calmness. I ask family members to record videos for the resident (E302–303). After the resident sees his/her son on the mobile phone, who does not talk to him, his/her attention is slightly diverted (E306–307).
Heading off a disaster using reverse psychology	(Collective restless behavior) When a group of older residents quarrel, pacification does not suffice. Therefore, we say: “Why not directly fight? Why quarrel.” The residents feel embarrassed. At this point, we ask them to sing, partake in activities, or exercise. This transition is sometimes better than pacification (B293–299). I say, “Wait! Wait! You can only quarrel after I say ‘Start’! The competition has not started yet, and winners can go overseas for a competition. It is a waste of your breath if you all quarrel now. After I say ‘Start,’ please quarrel all the way.” Then they laugh or even forget, or ask to sing or watch videos. Ke-liang Chu’s videos distract them, and they forget the entire incident (B300–305).
Theme 4 Non-suppression: subthemes and quotes	
A safe environment	When residents disassemble curtains or things and move them around, we first observe them without interrupting them as it makes them angry. Therefore, we first pacify other roommates, and “let the resident move around a little.” When the resident is exhausted, he/she sits quietly. We then show concern for him/her and switch on his/her favorite television channel (A353–360). For residents who keep wandering around, we allow them to do so. When they become tired, they sit down, point at the air and scold, and then resume walking. We follow behind the resident to prevent him/her from falling or knocking onto objects, or hitting other older residents. We just need to ensure their safety. This is how we handle them (C194–98).
Going with the flow	(Clamoring to go home) We open the door for them; telling them that it is not possible agitates them further (E104–106). I fix a time with the resident, telling him/her that I would take him/her home at that time (E107). (Rushing for lifts) There is an elevator in the unit whose doors do not open. We tell the resident that the elevator doors will open later, and bring him/her a chair to wait in the elevator lobby. The resident sits there quietly, awaiting the elevator doors to open (B107–113). (Using onsite facilities and equipment to go with the flow)
Cold treatment	Sometimes, we are unable to pacify an agitated resident. We just let him/her walk around. Additionally, we have other tasks to complete; therefore, we just let him/her be! Once we are free, we attempt to pacify him/her again. After a while, his/her agitation subsides, and he/she forgets what had just happened (A160–164). (Agitated behavior in two or more people) We separate them and take them away from the scene to avoid affecting others. Sometimes, we take them for a walk, which leads to a slight improvement (B140–144). (When aggressive behavior occurs) I take the resident to an open space and ask him/her to calm down. This is because he/she may shout if left at the scene, and hit us if we approach him/her. We are also afraid that he/she will fall; therefore, we first take him to an open space (D074–D076).
Theme 5 Collaboration: subthemes and quotes	
Between colleagues	When an older resident becomes agitated and my methods prove ineffective, I seek help from my colleagues. They switch with me and accompany the resident on a walk to soothe his/her emotions (D035–037).
Support from residents	When several residents simultaneously become agitated, first we take the leader away. Often, other older residents without SS around the scene can play a role in the pacification. Usually, we arrange for them to chat and perform activities together, such as watching television. This helps pacify residents with SS (B248–252).
Support from non-clinical coworkers	A recently admitted, highly aggressive older resident was unmanageable for even 4–5 people. We sought assistance from security guards and administrative staff (D108–111). In our entire institution, other external administrative staff could help. One day after dusk, an older resident kept wanting to go out and I had no choice but to take him on a big round around the park. However, he knew the location of the doors; therefore, we asked the security guard to lock the main door (D112–122).
Theme 6 Continuity of meeting: subthemes and quotes	
Medication adjustment	Excluding sporadic cases, when family members called or the resident was brought out of the institution, if the frequency of problematic behavior increases even though there is no major change to his/her daily life, we verify the records, discuss, and may recommend the physician to administer drugs (C088–095). For residents with more severe symptoms, we observe for a period of time to ascertain whether the dose be increased. If this was an occasional incident, we would not increase the dose as these mood-regulating drugs tend to cause them to fall (D093–095).
Enhancing safety precautions	Most of our efforts aim to prevent falls. These days, we rarely encounter emotional agitation. Unless the resident becomes agitated or is a hazard, the probability of using restraints is negligible (B447–449). A while ago, an older resident wanted to climb the door; I restrained the resident with the help of two males. We knew about the resident’s condition; therefore, we temporarily restrained him for safety (A414–419).

SS = sundown syndrome

### Theme 1: self-preparation

This theme reflects frontline staff preparation for caring for PLWD with SS in the intra-individual dimension. It illustrates four subthemes: self-awareness, familiarity with residents and establishing trust, identification of prodromal symptoms, and exploration of possible causes.

The subtheme *self-awareness* emphasized that the SS of residents affected the emotions of nurses and nurse aides, specifically as they felt exhausted later in the day. Some nurses and nurse aides highlighted the importance of self-awareness and emotional management. *Familiarity with residents and establishing trust* was identified as the first step in establishing trust among residents, which was beneficial to their care of residents with SS. *Identification of prodromal symptoms* can have some advantages on the evidence of issues related to SS by facilitating the adoption of preventive strategies early. *Exploration of possible causes* can help nurses and nurse aides realize the residents with SS because their restless behavior or emotional responses must result from reasons such as expressing their needs.

### Theme 2: pacification

This theme reflects an effective optimal strategy for caring PLWD with SS in the inter-individual dimension. It illustrates four subthemes: listening, empathy, little white lies, and role play.

*Listening* made nurses and nurse aides understood that the residents had SS as a result of unmet needs. *Empathy* made nurses and nurse aides knew that some residents are concerned about their families even though the residents with SS are confused. *Little white lies* outlined a technique that prioritized the individual needs, preferences, and values of residents with SS to provide more effective and compassionate care. *Role play* highlighted another effective technique for dealing with SS residents in certain situations. Nurses and nurse aides can distract, reassure, and pacify residents experiencing confusion, anxiety, or paranoia using role-playing to manage challenging SS scenarios.

### Theme 3: diversion

This theme reflects another optimal and effective approach in the inter-individual dimension. It illustrates six subthemes: arranging individual activities, pandering to their interests, changing the scene, accompanying dedicated staff, using technology, and heading off a disaster using reverse psychology.

*Arranging individual activities* outlined the importance of understanding and responding to the unique needs of SS residents. By engaging in calm activities, nurses and nurse aides can help manage SS symptoms and improve quality of life. *Pandering to their interests* highlighted the importance of individualizing care for older residents with dementia and providing activities and stimuli that match their preferences and interests. *Changing the scene* outlined the guidance of residents to a different environment, away from the stimulus causing their SS symptoms, which specifically focused on managing their emotions and reducing behaviors such as agitation, hallucinations, and wandering. *Accompaniment by dedicated staff* emphasized the difficulties of understanding the thoughts and intentions of the residents based on their behavior if they had SS; the process of accompanying and engaging them in activities can divert their attention and help them feel more comfortable. *Using technology* outlined an innovative approach to divert residents’ attention. *Heading off a disaster using reverse psychology* highlighted the rationale of redirecting the residents’ attention (e.g., using humor). Instead of attempting to pacify residents, the residents’ attention was redirected toward more positive activities that could distract them from the source of their agitation.

### Theme 4: non-suppression

This theme reflects the third type of optimally effective approach in the inter-individual dimension. It illustrates three subthemes: a safe environment, going through the flow, and cold treatment.

*A safe environment* could help nurses and nurse aides take steps to minimize disruptions to their routines if the residents had SS. *Going with the flow* outlined creative approaches instead of telling residents that it was not possible to leave. However, it is important to ensure the safety of residents and monitor their behavior. *Cold treatment* sometimes works. As nurses and nurse aides faced situations in which residents showed aggressive or other behaviors without any reason, taking residents away from the scene and changing the environment, if necessary, ensured that their behavior did not escalate or cause any injury.

### Theme 5: collaboration

This theme reflects the importance of teamwork and is planned in the organizational dimension. It illustrates three subthemes: between colleagues, support from residents, and support from non-clinical coworkers.

*Between colleagues* highlighted the importance of seeking help from colleagues, especially when managing agitated behaviors in older residents with SS. *Support from residents* was an important experience shared by nurses and nurse aides who emphasized the effectiveness of identifying the leader and using peer-to-peer interactions to pacify residents with SS. *Support from non-clinical coworkers* outlined the importance of team cooperation. Engaging in nonclinical roles in a care team has several advantages. Some nurses and nurse aides sought assistance from other staff members such as security guards and administrative staff.

### Theme 6: continuity of meeting

This theme reflected the importance of planning and communication in caring for PLWD with SS. It illustrates two subthemes: medication adjustment and enhancing safety precautions.

*Medication adjustment* was pointed out. Some nurses and nurse aides focused more on managing residents’ behaviors through medication rather than addressing underlying issues or providing non-pharmacological interventions. *Enhancing safety precautions* outlined concerns about ensuring the safety of residents, particularly by preventing falls. The focus was on balancing the need to ensure the safety of the residents with the desire to avoid the use of restraints as much as possible.

## Discussion

This exploratory qualitative study examined PCC-related responsive care practices and management experiences in DSCUs when residents develop SS. In the case of the units recruited for this study, there are more nurse aides than nurses. According to the regulations of the Taiwanese Establishment Standards of Senior Citizens’ Welfare Institutions, one nurse must be appointed for every 20 residents in each DSCU. During the daytime, one nurse aide must be assigned for every three residents, and during the nighttime, one nurse aide must be assigned for every 15 residents. Nurses and nurse aides are well positioned in the DSCUs and nurse aides may be in the majority. Nurses and nurse aides described special SS scenarios requiring significant care, including wandering, seeking exits, continuously clamoring to go home/search for family members, and repetitive speech or behavior. Generally, patients showed various symptoms [3, 18] and showed a heterogeneous presentation [2]. Some possible causes of SS were observed by the participants, including visits by family members and physical environmental problems. Other causes of SS include circadian rhythm disruption, decreased cognitive reserve, and fatigue experienced in one day [2, 5].

Given the challenges of caring for PLWD with SS, we found that the PCC model conducted with different practices that align with the respective residents’ unmet needs may improve the quality of life of PLWD and decrease the behavioral and psychological symptoms of dementia [11, 23]. At the individual level, when supporting PLWD with SS, self-awareness can protect the nurses and nurse aides from negative emotions. Establishing familiarity and trust with PLWD beforehand, a point mentioned by the nurses and nurse aides, is consistent with the PCC framework. Understanding the daily lifestyles and life stories of the residents enables a focus on each individual, not the disease, and helps ascertain the current physical awareness and potential of PLWD [12]. A process of self-preparation is necessary for nurses and nurse aides to conduct PCC and address the challenges of PLWD with SS. In Taiwan, nurses and nurse aides are required to undergo 20 h of training in dementia care. Both these professionals receive 2−4 h of training courses annually in addition. SS identification and steps in self-preparation can be part of the training, which would be beneficial for these frontline care providers for determining possible causes of the condition and sharing the understanding with PLWD.

At the inter-individual level, some practices to support PLWD with SS aligned with the 12 types of positive interactions identified by Kitwood [6, 8], intending to fulfil the psychological needs of PLWD. Pacification is the initial stage with nurses and nurse aides in addition to the PLWD. Validation therapy, a low-investment practice that respects the feelings of PLWD, is used for PCC with minimal negative effects. However, nurses and nurse aides must be prepared to alleviate their feelings; otherwise, validation therapy may exacerbate their suffering [8]. Deceptive interactions between nurses, nurse aides, and residents with SS can have multiple ramifications, both positive and negative. Moreover, telling few white lies or deception was identified in the study. In Eastern cultures, a strong emphasis is placed on family relationships and maintaining harmony by avoiding negativity and emotional outbursts. Nurses and nurse aides prefer telling white lies when PLWD with SS want to return home or seek their family members. This practice can reduce the negative emotions of residents with SS. However, perspectives of telling white lies are inclusive, and a prior Western study suggests that it is acceptable when there is an established prior relationship with the resident [24].

We found that diversion and non-suppression are required in PCC practice for PLWD with SS. However, this does not entail reaffirming the authority of the nurses and nurse aides; rather, it is about satisfying the needs of PLWD through specific approaches. These strategies alleviate physical and mental discomfort in PLWD, thereby lessening physical and mental exhaustion in nurses and nurse aides. Practice recommendations for PCC demonstrate that respect should be accorded to residents, and equal respect should be felt in interpersonal relationships [9]. Residents’ individuality should be prioritized in the care relationship, emphasizing interactions and attention over tasks. Moreover, in accommodation institutions, nurses and nurse aides can take residents away from the site where SS is induced in consideration of other residents. To fulfil the needs of PLWD, a safe space and environment are required [6]. This approach contrasts that of previous care settings in Taiwan’s long-term care institutions where residents were restrained to ensure their safety, resulting in a form of deprivation [25]. Providing personalized care, supporting personalized options for PLWD, and maintaining respect are vital for establishing interpersonal relationships [9].

The organizational-level provision of PCC requires a top-down and bottom-up approach. While nurses and nurse aides are skilled in the delivery of PCC among PLWD, the entire organization must also be supportive, which would be beneficial to the quality of life of PLWD [26]. Collaboration with colleagues and residents helps manage SS, while non-frontline teams can assist with highly agitated residents. Teamwork forms the core of good care [9]. Additionally, interdisciplinary meetings facilitate diverse discussions and offer suggestions for managing challenging scenarios of care. However, from the perspective of the organization or leaders, flexible adjustment of care manpower, training volunteers to provide services during these periods, encouraging factors to visit during these periods, or using supportive physical tools and environments (e.g., light adjustments and space arrangements) can be considered to target possible causes.

In the Western PCC framework, “love” is the core value [6], but people in Asian cultures express “love” with fewer words, in a more implicit manner. In our results, “commitment” is a core value and more appropriate in PCC in Asian cultures, noted at the intra-individual, inter-individual, and organizational levels. Applying PCC concepts rooted in Asian cultures, the nurses and nurse aides in our study are well positioned to describe what is actually ongoing in direct resident care and how to actualize PCC practices among PLWD with SS in these contexts. The study provides some insights for managers, supervisors, and practitioners implementing PCC among PLWD with SS in Asian DSCUs; indeed, such stakeholders should know what professional caregivers experience and how they perceive PCC in practice, to facilitate improved care and support.

There are some limitations to this study. First, data were not collected through nursing records or case analyses because experienced institutional nurses and nurse aides can provide good SS-related care information. As SS is similar to other neuropsychiatric symptoms, there is an increasing consensus on the use of non-drug methods as the first-line treatment, and drug treatment is restricted to PLWD who do not respond to non-drug methods [18].

## Conclusion

This study adds to the literature by describing the PCC-based care experiences of DSCU nurses and nurse aides supporting PLWD with SS. It sheds light on the management methods that can be adopted when problematic SS behaviors occur in PLWD, provided reference for nurses and for developing professional training materials. It can contribute to an improved quality of care provided by nurses and nurse aides who take care of older adults with dementia and SS.

### Electronic supplementary material

Below is the link to the electronic supplementary material.

Supplementary Material 1

## Data Availability

The datasets generated and/or analyzed during the current study are not publicly available due to the sensitive nature of the data [individual privacy could be compromised] but are available from the corresponding author on reasonable request.

## Abbreviations

PLWD: people living with dementia
SS: sundown syndrome
PCC: person-centered care
DSCUs: dementia special care units
