# Supplementary material for: Experiences of person-centered care for sundown syndrome among nurses and nurse aides in dementia special care units: a qualitative study
Source: BMC Nurs. 2023 Nov 17;22:435. doi: 10.1186/s12912-023-01598-x (PMC10655402; doi:10.1186/s12912-023-01598-x)
Supplement: Supplementary file 1 — Supplementary Material 1 [file 12912_2023_1598_MOESM1_ESM.docx]

Additional Table 1. Our study’s example of the analysis process

| *Meaning unit* | *Code* | *Subtheme* | *Theme* |
| --- | --- | --- | --- |
| Often, residents may become confused. When I hear that an older woman is searching a scarf, I think: “Oh no, here she comes again, and know that today is going to be a bad day.” | Emotion affected by residents with SS | Self-awareness | Self-preparation |
